# Network analysis of core factors related to non-suicidal self-injury in adolescents with mood disorders

**DOI:** 10.3389/fpsyt.2025.1557351

**Published:** 2025-04-29

**Authors:** Wenyan Zhang, Xiaohui Song, Xianbin Wang, Zhongliang Jiang, Yuebing Zhang, Yonghua Cui

**Affiliations:** ^1^ Department of Psychiatry, Beijing Children’s Hospital, Capital Medical University, National Center for Children Healthy, Beijing, China; ^2^ Out-patient Department, Jining NO.2 People’s Hospital, Jingning, Shandong, China; ^3^ Department of Psychiatry, Shandong Daizhuang Hospital, Jingning, Shandong, China

**Keywords:** non-suicidal self-injury, mood disorders, adolescents, machine learning, network analysis

## Abstract

**Introduction:**

Adolescents with mood disorders are at an exceptionally high risk for non-suicidal self-injury (NSSI); however, the understanding of the core factors underlying this vulnerability remains limited. This knowledge gap significantly hampers the effectiveness of targeted prevention and intervention strategies.

**Methods:**

A total of 263 adolescents with mood disorders completed a series of self-report surveys, covering demographic, personal, and social factors related to NSSI. We first used least absolute shrinkage and selection operator (LASSO) regression to identify the core related factors. Then, we employed network analysis to construct the network structure of these core factors.

**Results:**

Our findings indicate that depressive and anxiety symptoms are the strongest influencing factors for NSSI among adolescents with mood disorders. Life events and the specific functions of NSSI are identified as personalized factors within this group. Additionally, objective social support and education level emerged as potential protective factors against NSSI. These factors are not independent but interact with each other.

**Conclusion:**

By identifying and intervening in these key factors, more effective prevention strategies and personalized treatment plans can be developed, ultimately improving the quality of life and psychological well-being of adolescents with mood disorders.

## 1 Introduction

Non-suicidal self-injury (NSSI) refers to deliberate self-inflicted harm without suicidal intent, frequently observed among adolescents (1). A comprehensive review of global adolescent populations reports an average lifetime prevalence of NSSI at 18.0% (2). The relationship between mood disorders and NSSI is significant (3), with studies indicating that the prevalence of NSSI in individuals with mood disorders is as high as 62%, far exceeding the average prevalence among adolescents (4). This suggests that adolescents with mood disorders are at a high risk for NSSI. Despite extensive research on the prevalence and associated factors of NSSI, studies specifically targeting NSSI in adolescents with mood disorders remain limited. The interaction between emotional dysregulation and other psychological factors may result in unique patterns of NSSI in this population.

The strong comorbidity between NSSI and adolescent mood disorders can be understood through three foundational theoretical frameworks. First, Linehan’s biosocial theory of emotion dysregulation (5) suggests that a biological predisposition to emotional intensity, when coupled with an invalidating environment, fosters maladaptive coping mechanisms such as NSSI. This aligns with neuroimaging findings indicating altered prefrontal-limbic circuitry in mood disorders (6), which may heighten emotional reactivity and impair regulatory capacity. Second, Joiner’s interpersonal theory (7) expands on this by highlighting the roles of thwarted belongingness and perceived burdensomeness, both common in depressed adolescents, in fostering self-punitive motivations for NSSI. Third, Nock’s integrated theoretical model (8) synthesizes these perspectives, proposing that NSSI serves four key reinforcing functions in psychopathology: automatic negative reinforcement (e.g., alleviating emotional distress), automatic positive reinforcement (e.g., self-punishment), social negative reinforcement (e.g., avoiding external demands), and social positive reinforcement (e.g., eliciting care and support). Collectively, these theories underscore the dynamic interplay between trait-like vulnerabilities (such as heightened emotional reactivity) and state-dependent factors (such as acute mood episodes), which may explain why the risk of NSSI is 3.4 times higher during major depressive episodes compared to euthymic periods (9).

Current research has identified several factors related to NSSI in community populations, including demographic, individual, and social factors (10). First, factors such as sex, age, and education level are significant demographic factors for NSSI. Studies indicate a higher prevalence of NSSI among female adolescents, with variations across different age groups (11, 12). Lower educational attainment may lead to a lack of effective stress coping strategies, increasing the risk of NSSI (13). Second, depression, anxiety, gaming addiction, and coping styles are common individual factors. Depression and anxiety are significant predictors of NSSI, as individuals may use NSSI to alleviate emotional distress and pressure when facing emotional dysregulation (14). Behavioral addictions such as internet gaming disorder are also related to NSSI, as these individuals often use gaming to escape real-life problems, but unresolved emotional issues can lead to self-injury (15). Lastly, social support, life events, and broader family factors are important social factors. For instance, lack of social support and experiencing major life events increase the risk of NSSI (16, 17). Family factors such as parental education level, family relationships, and economic status significantly influence adolescents’ emotional support and behavior patterns (18). However, there is a lack of research specifically focusing on adolescents with mood disorders. While many studies have examined NSSI in the general adolescent population, they often fail to distinguish those with mood disorders, who may exhibit distinct risk factors and behavioral patterns. Furthermore, the multifaceted nature of these factors makes it challenging to accurately identify the most critical ones.

The emotion regulation model supports the unique patterns of NSSI that may appear in individuals with mood disorders. This model posits that difficulties in emotion regulation exacerbate the symptoms of mood disorders and interact with other psychological factors such as cognitive distortions, maladaptive coping strategies, and social environmental stressors. This interplay can lead to complex and varied behavioral patterns, including NSSI (16, 17, 19). Therefore, due to the interaction between emotional dysregulation and other psychological factors, this population may exhibit unique patterns of NSSI. However, current research predominantly explores the factors related to NSSI in isolation, without considering the interactions among these factors.

In recent years, advanced analytical methods have been increasingly applied to identify NSSI risk factors with greater precision. Machine learning models, such as support vector machines and neural networks, have shown promise in predicting NSSI, but they often lack interpretability and may require large datasets for optimal performance (20, 21). Additionally, studies using longitudinal designs have provided deeper insights into the temporal dynamics of NSSI, highlighting the role of early-life stress and chronic emotional dysregulation (22, 23). However, most prior research has examined NSSI-related factors in isolation, without systematically assessing their complex interrelationships.

To address these issues, we employed LASSO regression and network analysis. LASSO regression is a highly effective statistical method for handling high-dimensional datasets. It selects the most influential variables in the model while excluding irrelevant or redundant ones (24). This method is frequently used for dimension reduction of complex related factors (25–29). Therefore, we utilized LASSO regression to identify the factors with the strongest influence on NSSI. Compared to other machine learning methods such as neural networks (30), LASSO regression offers greater interpretability and is less prone to overfitting when dealing with relatively small sample sizes. Neural networks, while powerful in detecting complex patterns, often require large datasets and extensive computational resources, making them less practical for studies with moderate sample sizes and high-dimensional psychological data. Moreover, neural networks typically operate as “black box” models, limiting their ability to provide clear insights into the specific contribution of each predictor to NSSI. In contrast, LASSO regression allows for direct identification of the most relevant predictors, facilitating clearer theoretical interpretations in psychological research. In the context of mental disorders, symptoms are typically not isolated but interconnected through complex relational networks. Network analysis offers an intuitive approach to reveal the interrelations among symptoms (31, 32). Unlike traditional statistical models that assume linear relationships (33), network analysis provides a graphical representation of how NSSI-related factors interact dynamically. This method enables us to identify central factors within the network that may serve as key intervention targets, thereby offering a more comprehensive perspective on the mechanisms underlying NSSI. Consequently, compared to previous studies, traditional regression methods primarily identify associations, whereas LASSO regression enhances factor selection by handling high-dimensional data, and network analysis reveals the underlying structure of these relationships. This makes our study particularly well-suited for identifying key predictive factors and their interconnections.

To bridge this gap, our study focuses on examining the network patterns of core factors related to NSSI in adolescents with mood disorders. We hypothesize that certain psychological and social factors will be core predictors of NSSI in adolescents with mood disorders. Specifically, our research involves: (i) utilizing LASSO regression to reduce the dimensionality of the complex related factors of NSSI and identify the core factors; (ii) employing network analysis to model the core factors related to NSSI.

## 2 Materials and methods

### 2.1 Participants & procedure

**Figure 1:**
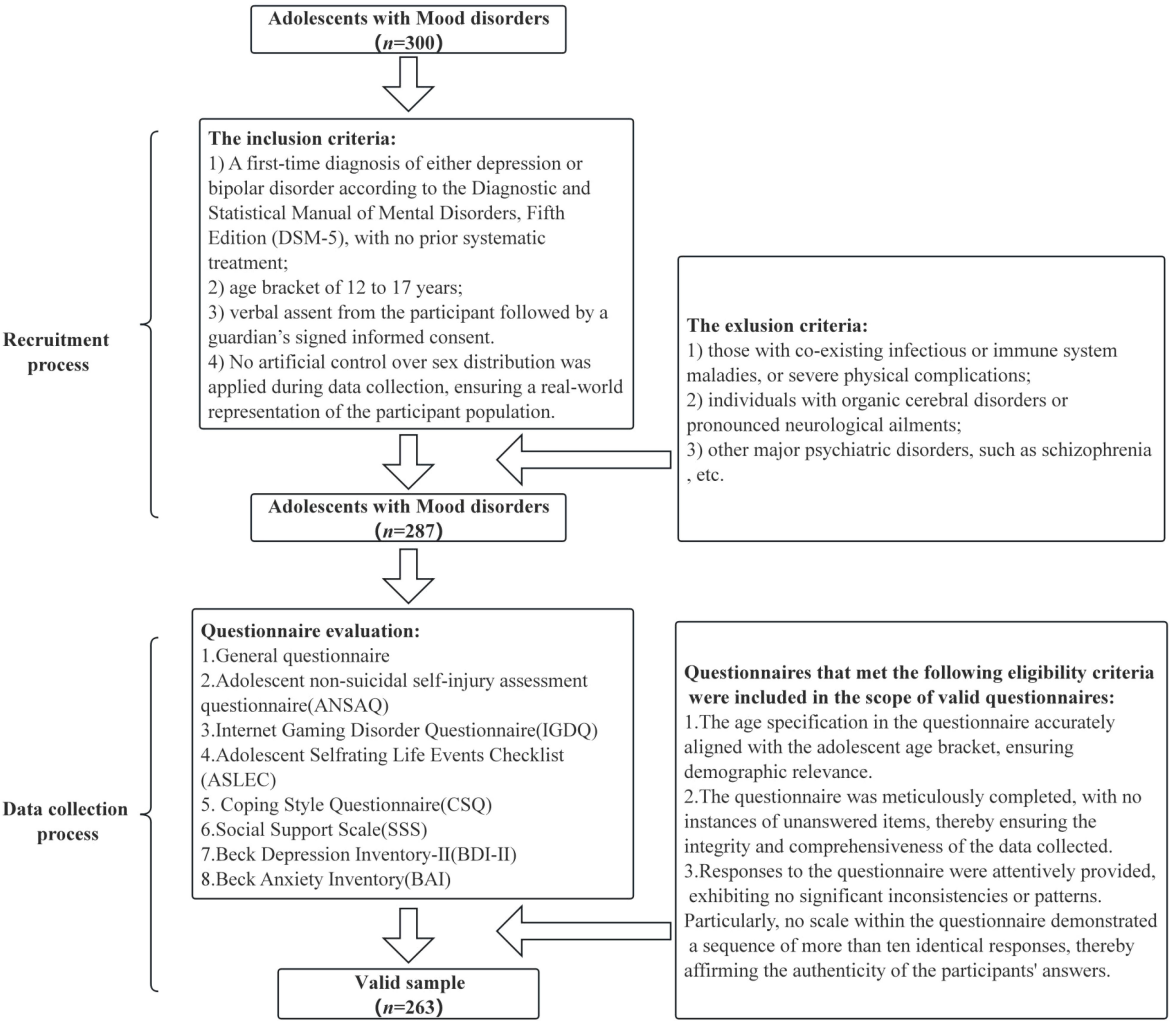
Recruitment and data collection process of adolescents with mood disorder.

Participants were sourced from the Child Psychiatry Outpatient Clinic of a psychiatric facility in southwestern Shandong, China, from August to December 2020. This region was chosen for its strong cultural representativeness, providing valuable insights into NSSI within a culturally relevant context. For inclusion, participants needed to satisfy the following criteria: 1) A first-time diagnosis of either depression or bipolar disorder according to the Diagnostic and Statistical Manual of Mental Disorders, Fifth Edition (DSM-5) (34), with no prior systematic treatment; 2) age bracket of 12 to 17 years; 3) verbal assent from the participant followed by a guardian’s signed informed consent; 4) No artificial control over sex distribution was applied during data collection, ensuring a real-world representation of the participant population. On the flip side, exclusions encompassed: 1) those with co-existing infectious or immune system maladies, or severe physical complications; 2) individuals with organic cerebral disorders or pronounced neurological ailments; 3) other major psychiatric disorders, such as schizophrenia, etc. **Figure 1** elaborates on the recruitment methodology and data acquisition process. Our investigation was anchored in the tenets of the Helsinki Declaration and procured approval from the Ethical Board of Shandong Daizhuang Hospital.

From the 287 participants who satisfied the study criteria, 263 valid samples were ultimately retained for analysis. Of these, 233 were diagnosed with depressive disorders, while the remaining participants had bipolar disorder. The sample comprised 87 males and 176 females, with an average age of 15.12 ± 1.606 years.

### 2.2 Data collection procedure

Participants were recruited during their clinical visits, and trained clinicians and research assistants conducted structured clinical interviews and administered standardized questionnaires to assess demographic and clinical characteristics. To ensure data validity and reliability, only questionnaires meeting the eligibility criteria were included. These criteria included age consistency, completeness, and response consistency. Detailed criteria are presented in **Figure 1**. All collected data were de-identified, with each participant assigned a unique study ID. Personal identifying information was stored separately from research data and was accessible only to authorized research personnel. The study followed strict confidentiality protocols in accordance with ethical guidelines set by the Ethical Board of Shandong Daizhuang Hospital, ensuring participant anonymity and data protection.

### 2.3 Measures

#### 2.3.1 General situation questionnaire

The General Situation Questionnaire, a tool we developed in-house, captures pertinent demographic details such as diagnosis, sex, age, in addition to family, school, and other relevant factors. Comprehensive details are provided in **Supplementary Information 1** in **Supplementary Data Sheet 1**.

#### 2.3.2 Adolescent non-suicidal self-injury assessment questionnaire

The assessment of NSSI was conducted using the Chinese adaptation of the Adolescent Non-Suicidal Self-Injury Assessment Questionnaire (ANSAQ) (35), which traces its origins to the Functional Assessment of Self-Mutilation formulated by Lloyd et al. (36). The ANSAQ is bifurcated into two sections: behaviors related to self-injury and functions of self-injury. The behavioral segment comprises 12 items scored on a 4-point scale (0 = No, 4 = Always). Higher scores indicate an increased severity of NSSI behaviors. The functional segment has 19 items rated on a similar 4-point scale (0 = total nonconformity, 4 = complete compliance) and encompasses three dimensions: “egoistic social interaction”, “automatic-negative reinforcement”, and “emotional expression”. The reliability metrics for the ANSAQ are commendable, with Cronbach’s α values of 0.914 for the behavioral scale and 0.950 for the functional scale (35).

#### 2.3.3 Internet gaming disorder questionnaire

The Internet Gaming Disorder Questionnaire (IGDQ) utilizes an interview format, adhering to the diagnostic criteria set forth in DSM-5, encompassing nine distinct items. A diagnosis is inferred when individuals meet or exceed five of these criteria over a 12-month duration. While not formally recognized as a definitive diagnosis, the IGDQ serves as a pivotal benchmark for evaluating behaviors consistent with internet gaming disorder. The instrument’s reliability is robust, with a Cronbach’s α value of 0.910 (37).

#### 2.3.4 Adolescent self-rating life events checklist

The Adolescent Self-Rating Life Events Checklist (ASLEC), in its Chinese adaptation (38, 39), consists of items, scored on a 6-point scale ranging from 0 (never happened) to 5 (extremely severe). This checklist is organized into six distinctive dimensions: “relationship pressure”, “learning pressure”, “being punished”, “loss”, “adaptation problems”, and “other”. The ASLEC demonstrates notable reliability, with a Cronbach’s alpha coefficient reported at 0.849 (38, 39).

#### 2.3.5 Coping style questionnaire

The Coping Styles Questionnaire (CSQ) in its Chinese adaptation (40), comprises 62 items. Each item is scored on a dichotomous scale: “0 = no” and “1 = yes”. The instrument categorizes coping into six distinct dimensions: “problem-solving”, “self-blaming”, “help-seeking”, “fantasizing”, “avoidance”, and “rationalizing”. The reliability of each of the six subscales within CSQ is robust, with Cronbach’s α coefficients ranging between 0.700 and 0.770 (40).

#### 2.3.6 Social support scale

The Social Support Scale (SSS) in its Chinese adaptation (41) encompasses 17 items, scored on a 5-point scale ranging from 1 (conformance) to 5 (non-Conformance). It delineates social support into three primary dimensions: “subjective support,” “objective support,” and “support utilization.” The overall reliability of the SSS is commendable, with a Cronbach’s α coefficient of 0.821. The respective dimensions demonstrate reliability values ranging from 0.631 to 0.685 (41).

#### 2.3.7 Beck depression inventory-II

The Beck Depression Inventory-II (BDI-II), in its Chinese iteration (42), comprises 21 items, scored on a 4-point scale ranging from 0 (insensibility) to 5 (severe). The reliability of the BDI-II is robust, reflected by a Cronbach’s α coefficient of 0.930 (42).

#### 2.3.8 Beck anxiety inventory

The Beck Anxiety Inventory (BAI), in its Chinese adaptation (43), encompasses 21 items, evaluated on a 4-point scale spanning from 0 (insensibility) to 5 (severe). The BAI demonstrates commendable reliability, as indicated by a Cronbach’s α coefficient of 0.950 (43).

### 2.4 Analysis

The demographic characteristics and questionnaire scores were described as means and standard deviations (SDs) for continuous variables, or as counts and percentages for categorical variables. The skewness values of these factors ranged from -0.713 to 1.376, and the kurtosis values ranged from 2.1 to 4.055. Both skewness and kurtosis fall within the normal range, suggesting that the data approximately follow a normal distribution (44). The Pearson correlation coefficients among these factors were all below 0.7, indicating that while the factors are correlated, they are not redundant (45).

Dimensionality reduction using LASSO regression analysis was executed through the “glmnet”, “caret”, and “dplyr” packages in R software (version 4.3.1). “NSSIB” was designated as the dependent variable, and the remaining 49 factors associated with demographic, personal, and social factors were incorporated into the model as independent variables. We selected the λ value corresponding to one standard error of the minimum mean squared error (1se) to optimize model performance while retaining the most parsimonious set of predictor variables. The identified variables were then advanced for subsequent network analysis.

Network analysis was also executed in R version 4.3.1. We employed a Gaussian graphical model (GGM) to delineate the network (46), facilitating the examination of interconnections between network nodes. Throughout the network model estimation, the graphical least absolute shrinkage and selection operator (Glasso) algorithm was integrated with the extended Bayesian information criterion (EBIC) to yield a regularized partial correlation network that is both stable and interpretable (47).

Network integrity was appraised through a set of metrics: Initially, we deployed strength centrality to determine and quantify the significance of each node within the network. This metric was facilitated using the “qgraph” package in R (48). Subsequently, the network’s resilience was assessed through the “bootnet” package in R (49). We employed parametric bootstrap (comprising 1,000 iterations) to estimate edge weight precision, while a nonparametric bootstrap approach was used to ascertain node strength stability. To determine significant disparities between two edge weights or two node strengths, bootstrap resampling was conducted (1,000 iterations, with a significance threshold of α = 0.05).

## 3 Results

### 3.1 Basic information description

Comprehensive socio-demographic profiles and associated questionnaire assessment scores are presented in **Table 1**.

**Table 1:** Socio-demographic characteristics and questionnaire assessment score.

Variable	Total (*n* = 263)	
	*n*	%
Female sex	176	66.92
Diagnosis		
Depressive disorder	233	88.59
Bipolar disorder	30	11.41
	Mean	SD
Age	15.12	1.606
IGDQ	1.61	1.778
NSSIB	23.21	11.288
NSSIF1	20.4	8.447
NSSIF2	13.58	5.676
NSSIF3	11.19	4.71
ASLEC1	11.05	6.235
ASLEC2	10.24	5.873
ASLEC3	9.83	7.621
ASLEC4	2.9	3.568
ASLEC5	4.78	3.561
ASLEC6	6.66	4.513
CSQ1	5.65	3.384
CSQ2	6.34	2.857
CSQ3	3.78	2.638
CSQ4	5.85	2.102
CSQ5	5.62	2.265
CSQ6	4.92	2.039
SSS1	15.19	6.408
SSS2	20.09	7.009
SSS3	16.23	7.83
BDI	25.58	15.332
BAI	22.87	15.765

SD, standard deviation. ASLEC1 = “Interpersonal relationship”, ASLEC2 = “Academic pressure”, ASLEC3 = “Be punished”, ASLEC4 = “Lose”, ASLEC5 = “Health adaptation”, ASLEC6 = “Other life events”, CSQ1 = “Problem-solving”, CSQ2 = “Self-blame”, CSQ3 = “Seeking for help”, CSQ4 = “Fantasy”, CSQ5 = “Repression”, CSQ6 = “Rationalization”, SSS1 = “Subjective support”, SSS2 = “Objective support”, SSS3 = “Support utilization”.

### 3.2 LASSO regression analysis

**Figure 2:**
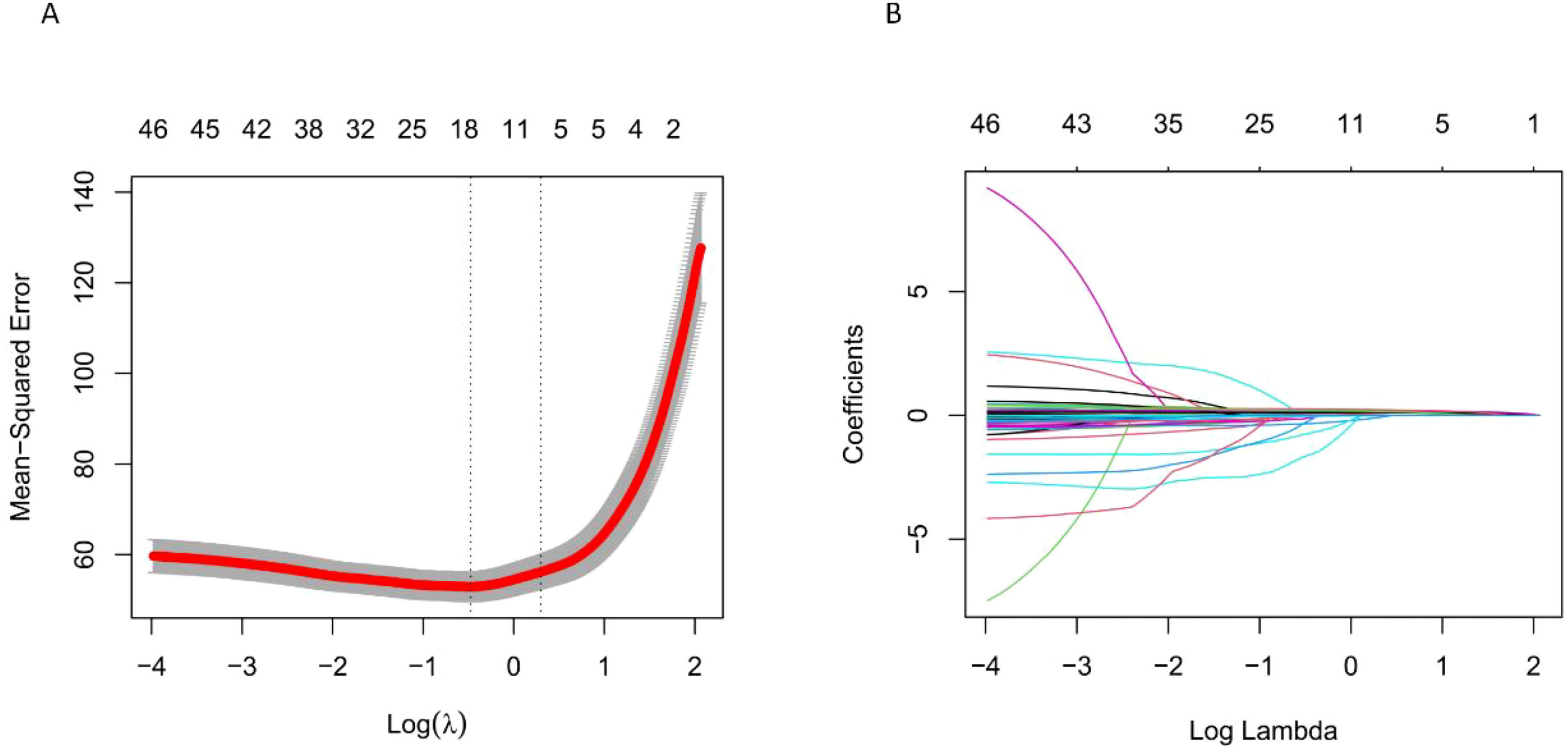
**(A)** shows the trend of mean squared error with changes in λ, and **(B)** shows the trend of regression coefficients with changes in λ. LASSO regression identified λ = 1.3476 at one standard error of the minimum mean squared error (1se), resulting in 8 selected independent variables.

From the initial 49 factors, our model identified 8 factors most closely related to the severity of non-suicidal self-injury behaviors (NSSIB): BDI (“depression”), BAI (“anxiety”), NSSIF1 (“egoistic social interaction”), NSSIF2 (“automatic-negative reinforcement”), ASLEC1 (“interpersonal relationship”), ASLEC6 (“other life events”), SSS2 (“objective support”), ITEM1 (“education level”). The resultant model outcomes are illustrated in **Figure 2**.

### 3.3 Network structure

**Figure 3:**
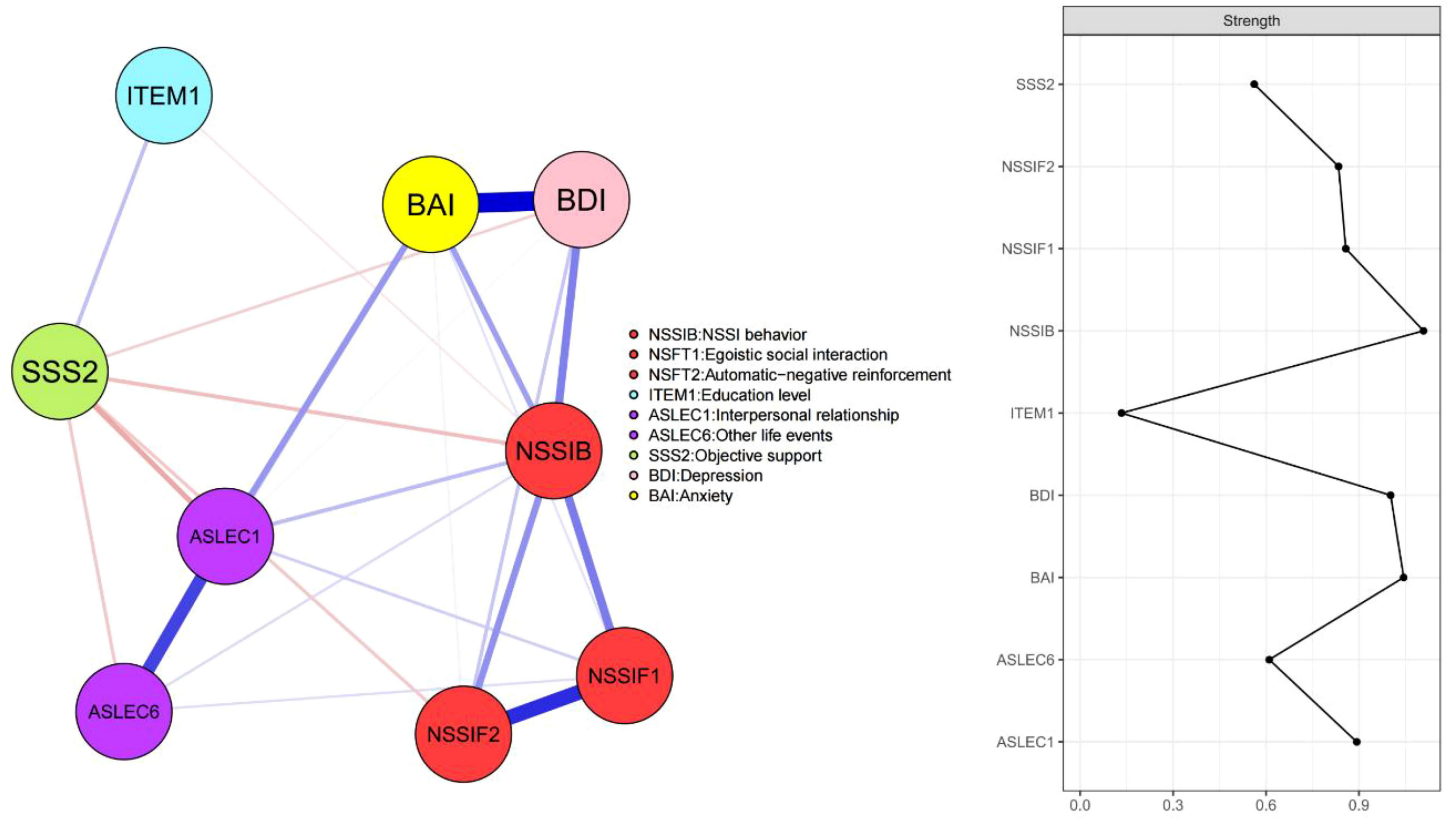
Network structure of core factors related to NSSI in adolescents with mood disorders. Within these network representations, blue lines signify positive associations, while red lines indicate negative ones. The magnitude of the correlation is visually captured by the line thickness.

**Figure 3** illustrates the results of a network analysis of core factors related to NSSI in adolescents with mood disorders. The network model shows that BDI (“depression”)—BAI (“anxiety”), NSSIF1 (“egoistic social interaction”)—NSSIF2 (“automatic-negative reinforcement”), and ASLEC1 (“interpersonal relationship”)—ASLEC6 (“other life events”) are the three strongest connections.

In terms of strength centrality, the node NSSIB (“NSSI behaviors”), indicating the severity of NSSI, exhibited the highest strength centrality. Following this, nodes related to emotional issues, BAI (“anxiety”) and BDI (“depression”), showed significant strength centrality. Next were nodes associated with personal factors: ASLEC1 (“interpersonal relationship”), NSSIF1 (“egoistic social interaction”), NSSIF2 (“automatic-negative reinforcement”), and ASLEC6 (“other life events”). Finally, the nodes ITEM1 (“education level”) and SSS2 (“objective support”), which are negatively correlated with the severity of NSSI, also demonstrated notable strength centrality.

Our findings revealed that the edge weights showcased commendable accuracy, as detailed in **Supplementary Information 2** in **Supplementary Data Sheet 1**. The node strength correlation exhibited a stability coefficient of 0.749, underscoring the robust stability of the network (**Supplementary Information 3** in **Supplementary Data Sheet 1**). Based on the resampling difference tests, there were notable disparities in the majority of the edge weights (**Supplementary Information 4** in **Supplementary Data Sheet 1**) and the centrality of node degrees (**Supplementary Information 5** in **Supplementary Data Sheet 1**) within the established network.

## 4 Discussion

The results of this study revealed the network of core factors related to NSSI in adolescents with mood disorders. This network model has the following main characteristics: First, the severity of emotional problems is the most central influencing factor. Second, life events and specific functions of NSSI are personalized factors within this population. Third, social support and education level may serve as potential protective factors against NSSI. Finally, these factors are not independent of each other but interact and influence one another. Compared to previous studies on NSSI risk factors (10), our findings place a stronger emphasis on core risk factors, providing valuable insights for more targeted interventions and treatments.

The severity of emotional problems is the strongest influencing factor for NSSI in adolescents with mood disorders. Individuals with mood disorders typically experience severe emotional issues such as depression and anxiety (50–52). On one hand, these emotional disturbances are major drivers of NSSI, as individuals may engage in self-injury to alleviate emotional pain or achieve temporary emotional relief (53, 54). Research shows that NSSI plays a significant role in emotion regulation, particularly in managing extreme negative emotions (55). On the other hand, the co-occurrence of depression and anxiety further complicates and intensifies emotional distress, thereby increasing the incidence of NSSI. The coexistence of depressive and anxiety symptoms not only exacerbates emotional pain but also weakens coping mechanisms, making individuals more likely to resort to NSSI as a coping strategy (56). Depression and anxiety are among the most critical risk factors for NSSI, further supporting the growing consensus on the emotion dysregulation model. This model posits that difficulties in regulating intense negative emotions play a central role in the development and maintenance of NSSI (5). The strong association between emotional distress and self-injury highlights the need for interventions targeting emotion regulation skills to reduce NSSI risk in adolescents with mood disorders.

Life events and specific functions of NSSI are personalized factors in individuals with mood disorders. First, interpersonal relationship problems are particularly prominent in this population, who often face challenges in maintaining and establishing healthy relationships (57, 58). Difficulties in social interactions can lead to feelings of loneliness and helplessness, thereby increasing the risk of NSSI (59–61). Negative social interactions and a lack of emotional support exacerbate emotional distress, making individuals more likely to engage in NSSI to cope with their emotional pain. Additionally, other life events include various negative experiences, such as the loss of a loved one, family conflicts, or academic pressure, which can trigger or worsen emotional issues, thus increasing the incidence of NSSI (62–64). In terms of specific functions of NSSI, egocentric social interactions reflect self-centered behavior in social settings, which is common among individuals with mood disorders. This behavior may lead to social rejection and feelings of isolation, further driving NSSI behavior. These individuals may engage in self-injury to gain attention or express emotional distress (53, 65). Automatic-negative reinforcement refers to using NSSI to relieve negative emotions and stress, a common coping mechanism in individuals with mood disorders who lack effective strategies to deal with emotional distress (53–55). Given that individuals experience life events and engage in NSSI for different reasons, these factors should be considered personalized and require targeted analysis. The significance of life events and the specific functions of NSSI in the occurrence of self-injury align with Nock’s integrated theoretical model of NSSI (8). This model emphasizes that individuals engage in self-injury for diverse and highly personalized reasons, with life events serving as key triggers.

Objective social support and education level may serve as potential protective factors against NSSI in adolescents with mood disorders. On one hand, higher education levels are generally associated with better cognitive abilities and problem-solving skills. Individuals with higher educational attainment often have more resources and knowledge to cope with emotional distress (66, 67), thereby reducing the incidence of NSSI. Education also helps to improve self-efficacy (68), enabling individuals to better face life’s challenges and stressors. On the other hand, objective support refers to tangible help and support from the social environment, such as family, friends, or social services. Research indicates that a strong social support network can provide emotional comfort and practical assistance, thereby reducing the risk of NSSI in individuals with mood disorders (69–71). Objective support can alleviate feelings of loneliness and helplessness, offering effective emotional regulation strategies that can substitute for NSSI as a coping mechanism (71). The finding that objective social support and education level serve as important protective factors against NSSI supports Joiner’s interpersonal theory, highlighting the crucial role of social relationships in preventing self-injurious behavior among adolescents facing emotional distress. According to this theory, strong interpersonal connections and a sense of belonging can reduce the risk of NSSI by mitigating feelings of isolation and despair (7). Additionally, higher education levels contribute to better interpersonal functioning, equipping individuals with the cognitive and social skills needed to navigate relationships more effectively (13).

In our study of NSSI in adolescents with mood disorders, we found that these factors do not exist independently; rather, they influence the occurrence and development of NSSI through complex interactions. Notably, through network analysis, we identified the complex connections between objective support, depressive moods, and NSSI. Depression was significantly identified as a precursor to NSSI, consistent with previous studies (72). Concurrently, we discovered that social support plays a mitigating mediating role between depression and NSSI (73), emphasizing its importance and potential preventive impact on NSSI among adolescents with mood disorders. Additionally, we observed interactions between adverse interpersonal life events, anxiety, and NSSI. Research indicates that while these negative life events directly influence NSSI, they also indirectly affect it through the mediating factor of anxiety (74). Therefore, our findings reveal the interactions between NSSI and its core related factors. Our study revealed that these risk factors are closely interconnected and collectively play a crucial role in the occurrence of NSSI, further validating the three fundamental theoretical models of NSSI discussed earlier (5, 7, 8). Importantly, our findings demonstrate that these models do not exist independently but rather interact dynamically, where the severity of one factor can influence others, potentially creating a vicious cycle that exacerbates NSSI risk.

While this study has its merits, it also possesses inherent limitations. Firstly, our research focused on a specific group—adolescents diagnosed with mood disorders. Therefore, our findings may lack generalizability, limiting the extrapolation of our conclusions. Secondly, given the cross-sectional design of this study, we were unable to delve into the causal relationships between NSSI and its associated factors. This means that while we were able to identify correlations between NSSI and its related factors, we could not determine if these factors lead to NSSI. Looking forward, we plan to conduct more extensive longitudinal studies, which will encompass a more diverse community population. Finally, as the sample in this study is primarily derived from China, the external validity of the findings may be limited, and the results may not be directly generalizable to populations in other countries or cultural contexts. Differences in culture, social environment, and genetic background may influence the research outcomes. Therefore, caution should be exercised when applying the findings to other regions or populations, considering these potential cross-cultural differences. Additionally, the risk factors selected for this study were chosen because they are prominent across various cultural contexts. Although we conducted an extensive review of relevant literature and designed the study based on these findings, it remains challenging to cover all possible risk factors comprehensively. Thus, there may still be other relevant risk factors that were not included, which could potentially influence the results.

## 5 Conclusions

In conclusion, this study constructed a core network of factors related to NSSI in adolescents with mood disorders. This network model reveals how emotional symptoms, life events, social support, and education level interact and collectively influence the occurrence and development of NSSI. By identifying and intervening in these key factors, more effective prevention strategies and personalized treatment plans can be developed. This approach can reduce the risk of NSSI, and improve the quality of life and psychological well-being of adolescents with mood disorders.

## Data Availability

The raw data supporting the conclusions of this article will be made available by the authors, without undue reservation.
