# Supplementary material for: Network analysis of core factors related to non-suicidal self-injury in adolescents with mood disorders
Source: Front Psychiatry. 2025 Apr 29;16:1557351. doi: 10.3389/fpsyt.2025.1557351 (PMC12069291; doi:10.3389/fpsyt.2025.1557351)
Supplement: Supplementary file 1 [file DataSheet1.docx]

**Supplementary information**

**Supplementary information 1.**

| The origin, meaning and assignment of the label | | |
| --- | --- | --- |
| Questionnaire | Label | Meaning |
| Adolescent Non-suicidal Self-injury Assessment Questionnaire (ANSAQ) | NSSIB | Total score of "self-injury behavior" dimension of ANSAQ |
|  | NSSIF1 | Total score of "Egoistic social interaction" dimension of ANSAQ |
|  | NSSIF2 | Total score of "Automatic-negative reinforcement" dimension of ANSAQ |
|  | NSSIF3 | Total score of "Emotional expression" dimension of ANSAQ |
| Internet Gaming Disorder Questionnaire (IGDQ) | IGDQ | Total score of Internet Gaming Disorder Questionnaire |
| Adolescent Self-rating Life Events Checklist (ASLEC) | ASLEC1 | Total score of "Interpersonal relationship" dimension of ASLEC |
|  | ASLEC2 | Total score of "Academic pressure" dimension of ASLEC |
|  | ASLEC3 | Total score of "Be punished" dimension of ASLEC |
|  | ASLEC4 | Total score of "Lose" dimension of ASLEC |
|  | ASLEC5 | Total score of "Health adaptation" dimension of ASLEC |
|  | ASLEC6 | Total score of "Other life events" dimension of ASLEC |
| Coping Style Questionnaire (CSQ) | CSQ1 | Total score of "problem-solving" dimension of CSQ |
|  | CSQ2 | Total score of "self-blame" dimension of CSQ |
|  | CSQ3 | Total score of "seeking for help" dimension of CSQ |
|  | CSQ4 | Total score of "fantasy" dimension of CSQ |
|  | CSQ5 | Total score of "repression" dimension of CSQ |
|  | CSQ6 | Total score of "rationalization" dimension of CSQ |
| Social Support Scale (SSS) | SSS1 | Total score of "Subjective support" dimension of SSS |
|  | SSS2 | Total score of "Objective support" dimension of SSS |
|  | SSS3 | Total score of "Support utilization" dimension of SSS |
| Beck Depression Inventory-Ⅱ(BDI-Ⅱ) | BDI | Total score of Beck Depression Inventory-II |
| Beck Anxiety Inventory (BAI) | BAI | Total score of Beck Anxiety Inventory |
| General Situation Questionnaire | DIAGNOSE | The diagnosis of the subjects |
|  | SEX | Sex of the subjects |
|  | AGE | Age of the subjects |
|  | ITEM1 | Education level |
|  | ITEM2 | Father's education situation |
|  | ITEM3 | Mother's education situation |
|  | ITEM4 | Harmonious parental relationship |
|  | ITEM5 | With whom to live |
|  | ITEM6 | Number of households |
|  | ITEM7 | Father is away from home |
|  | ITEM8 | Mother is away from home |
|  | ITEM9 | Family economy |
|  | ITEM10 | Living conditions |
|  | ITEM11 | severe calamity |
|  | ITEM12 | the only child |
|  | ITEM13 | Changes in the learning environment |
|  | ITEM14 | academic record |
|  | ITEM15 | Family members have low intelligence and poor living and labor abilities |
|  | ITEM16 | Family members are hyperactive and particularly mischievous when they are young |
|  | ITEM17 | Family members have difficulty studying since childhood and have poor exam scores |
|  | ITEM18 | Family members are grumpy, eccentric, and prone to impulsive behavior |
|  | ITEM19 | Family alcoholics or drug users |
|  | ITEM20 | Family members who violate the law |
|  | ITEM21 | Family history of mental illness |
|  | ITEM22 | Family suicide history |
|  | ITEM23 | Family epilepsy |
|  | ITEM24 | Severe chronic illness or disability in family members |
|  | ITEM25 | Parents marrying close relatives |

**
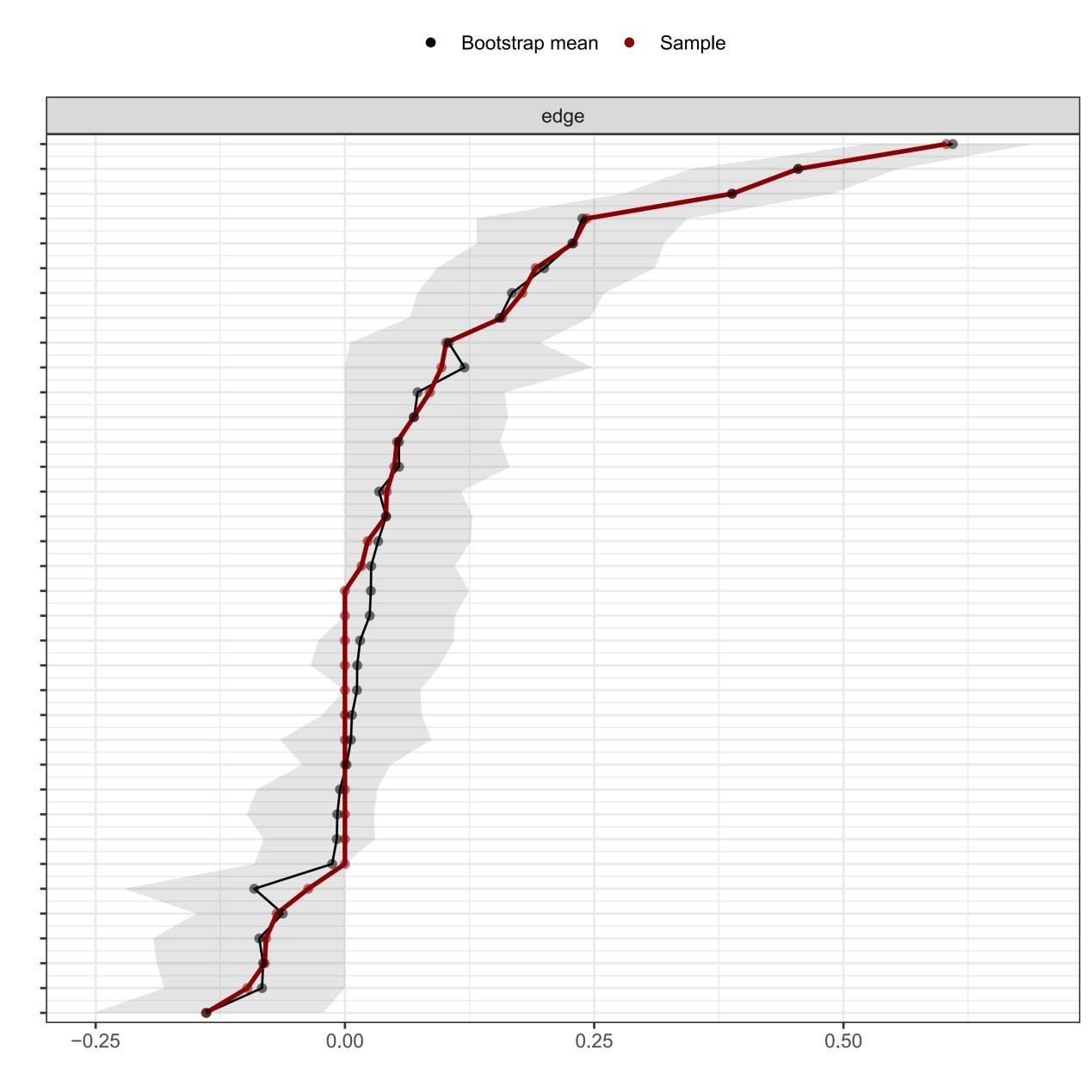
**

**Supplementary information 2.**

Accuracy of edge weights. The red lines represent the sampled edge weight values, while the gray shaded area indicates the bootstrap confidence intervals.

**
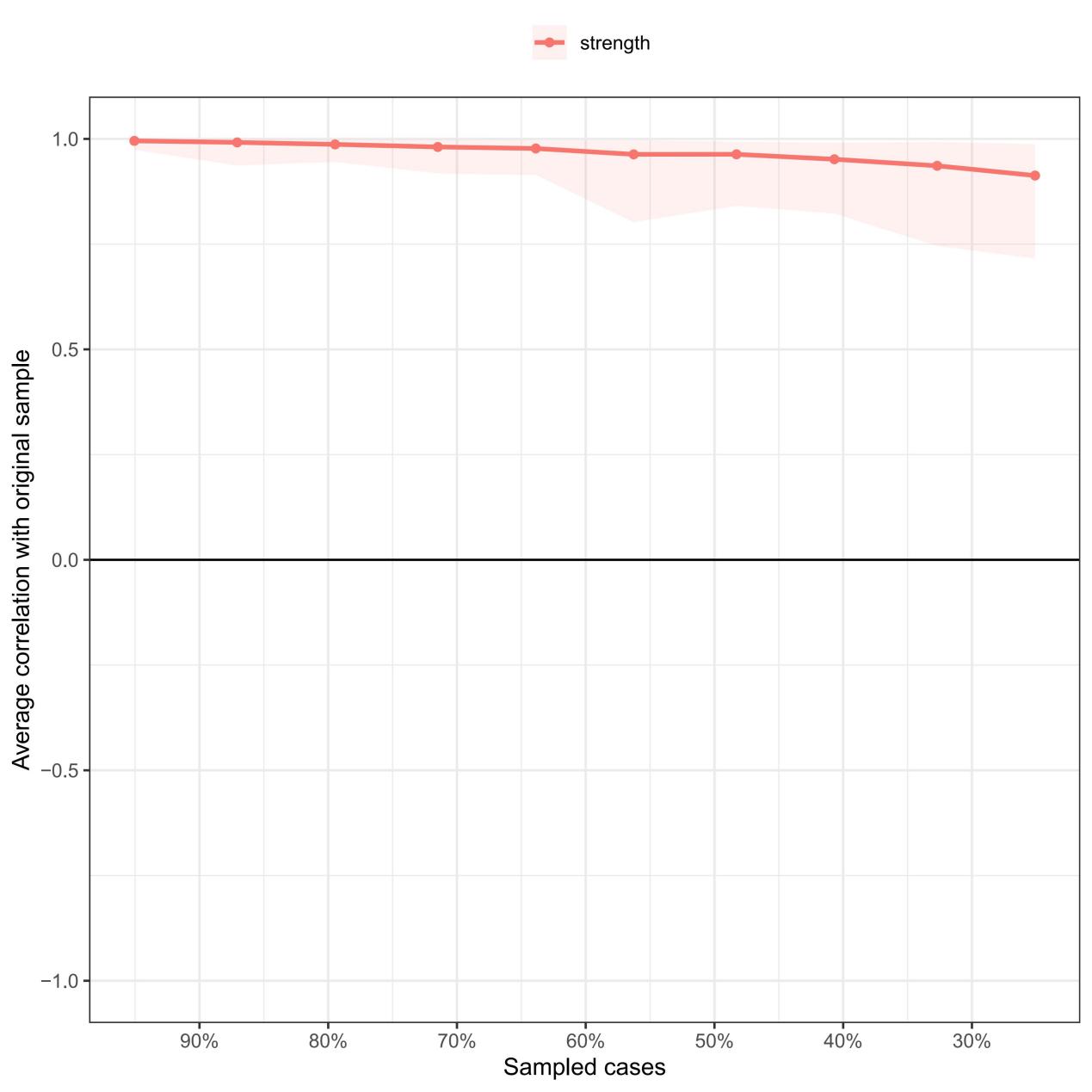
**

**Supplementary information 3.**

Stability of node degree centrality. The red line represents the average correlation between the original and resampled samples, while the pink shaded area represents the 2.5th and 97.5th percentile intervals


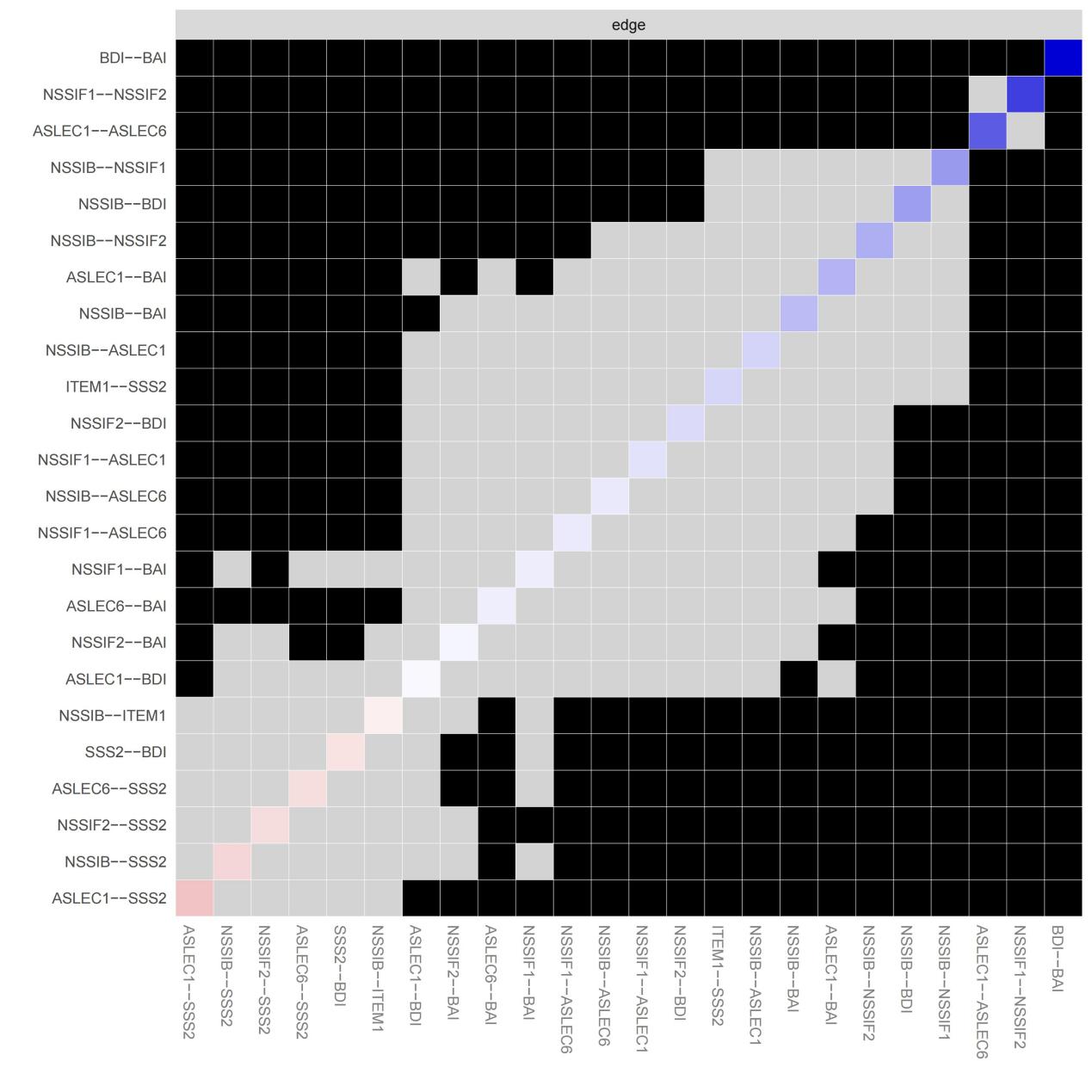


**Supplementary information 4.**

Difference test for edge weights. Black boxes indicate significant differences, while gray boxes indicate no statistical significance in the differences.


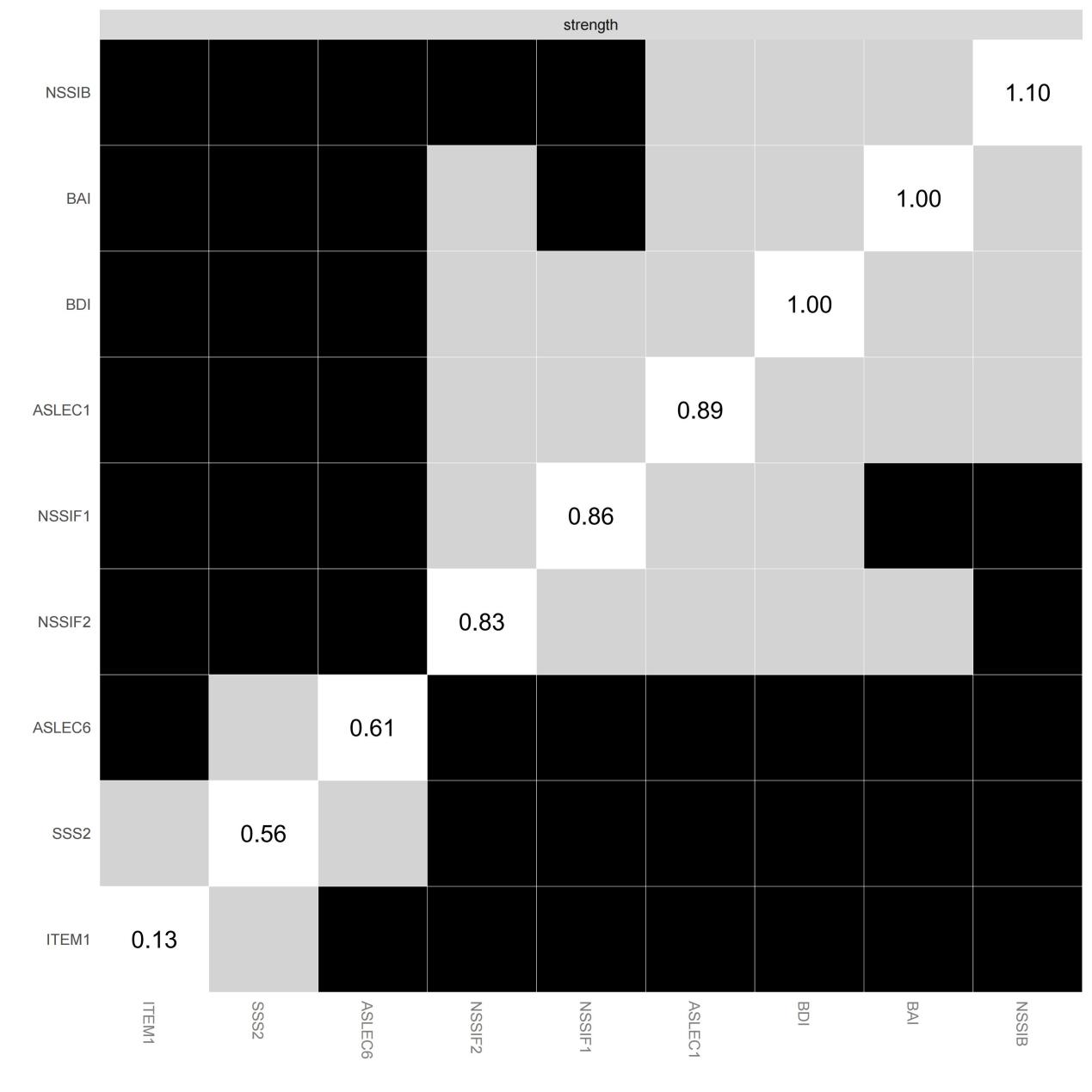


**Supplementary information 5.**

Difference test for node degree centrality. Black boxes indicate significant differences, while gray boxes indicate no statistical significance in the differences.
